# Gelatin methacryloyl and its hydrogels with an exceptional degree of controllability and batch-to-batch consistency

**DOI:** 10.1038/s41598-019-42186-x

**Published:** 2019-05-03

**Authors:** Mengxiang Zhu, Yingying Wang, Gaia Ferracci, Jing Zheng, Nam-Joon Cho, Bae Hoon Lee

**Affiliations:** 1 School of Ophthalmology & Optometry, Eye Hospital, School of Biomedical Engineering, Wenzhou Medical University, Wenzhou, Zhejiang 325027 China; 2 Wenzhou Institute of Biomaterials and Engineering, CAS, Wenzhou, Zhejiang 325011 China; 3 School of Materials Science and Engineering, Nanyang Technological University, Singapore, 639798 Singapore

**Keywords:** Biomaterials, Biomaterials - proteins

## Abstract

Gelatin methacryloyl (GelMA) is a versatile material for a wide range of bioapplications. There is an intense interest in developing effective chemical strategies to prepare GelMA with a high degree of batch-to-batch consistency and controllability in terms of methacryloyl functionalization and physiochemical properties. Herein, we systematically investigated the batch-to-batch reproducibility and controllability of producing GelMA (target highly and lowly substituted versions) via a one-pot strategy. To assess the GelMA product, several parameters were evaluated, including the degree of methacryloylation, secondary structure, and enzymatic degradation, along with the mechanical properties and cell viability of GelMA hydrogels. The results showed that two types of target GelMA with five batches exhibited a high degree of controllability and reproducibility in compositional, structural, and functional properties owing to the highly controllable one-pot strategy.

## Introduction

Protein-based hydrogels including collagen, fibrin, and Matrigel^®^, as natural extracellular matrices (ECM) analogues, have been widely investigated for various applications, like three-dimensional (3D) culture, tissue engineering, and regenerative medicine, because they are biocompatible and have excellent innate bioactive properties such as enzyme degradation and good control over cellular activities (adhesion, proliferation, migration, and differentiation)^1–4^. Matrigel^®^ is one of the most popular protein-based hydrogels^5–7^. Matrigel^®^ is a gelatinous protein mixture derived from Engelbreth-Holm-Swarm mouse sarcoma tumors and contains mainly proteins (laminin, type IV collagen, and entactin) and a small portion of proteoglycans and growth factors^5^. It has been found that Matrigel^®^ is a useful hydrogel for culture of cancer stem cells and stem cells, owing to its ability of improved cell-cell and cell material interactions^8,9^. However, Matrigel^®^ materials confront some challenges such as high batch-to-batch differences of their composition and poor control over mechanical properties, which can affect physiochemical and biological properties and elicit uncontrolled responses from cells^4,5^. In addition, Matrigel^®^ materials are practically not appropriate for clinical applications because they are originated from tumors.

Gelatin methacryloyl (GelMA) has been investigated as a potential alternative to Matrigel^®^ for 3D culture systems and bioapplications^10,11^. GelMA is an engineered gelatin-based material that has been proven to be versatile for tissue engineering, drug delivery, and 3D printing applications^12–19^. GelMA displays some important features such as biocompatibility, enzymatic cleavage (degradation in response to matrix metalloproteinases, MMPs), cell adhesion (arginine-glycine-aspartic acid, RGD sequences), and tailorable mechanical properties^14,15^. GelMA can be prepared through simple synthesis of gelatin with methacrylic anhydride (MAA), and its methacryloyl functionalization (or the degree of substitution (DS); the degree of methacryloylation (DM)) can be adjusted via a feed ratio of gelatin to MAA^20–23^. The DS of GelMA is one of the main factors that can influence biophysiochemical properties of GelMA and its photocured hydrogels. Recently, GelMA has been commercially available through some vendors such as Sigma-Aldrich. Therefore, there is a wide interest in developing effective methods to prepare GelMA with high reproducibility and controllability in terms of composition and biophysiochemical properties. Moreover, in most of the GelMA studies, only one batch of GelMA has been utilized for their research investigations^12,16,22,23^; a recent study dealing with three batches of various GelMA materials exhibited relatively high standard deviations in their methacryloyl functionalization and mechanical properties, potentially owing to their less controllable GelMA synthesis system and a subsequent batch-to-batch difference^24^. As far as we know, there is no systematic report regarding a batch-to-batch difference of GelMA and its hydrogel properties.

In this report, we investigated the batch-to-batch consistency and controllability of GelMA and its hydrogels. First, we prepared target GelMA samples (highly and lowly substituted versions: DS100_1~5 and DS60_1~5) with five batches via a one-pot synthesis strategy and then evaluated GelMA products in terms of synthesis, methacryloylation, protein structure, mechanical properties, degradation, and cell viability. Here, we demonstrate that target GelMA synthesized via the one-pot synthesis scheme showed a high degree of batch-to-batch consistency and controllability over their yields, degrees of substitution (DS), and secondary structure, as well as swelling, stiffness, degradation, and cell viability of their hydrogels.

## Results and Discussion

### Controllable preparation of highly and lowly substituted gelatin methacryloyl with five batches (DS100_1~5 and DS60_1~5)

**Figure 1:**
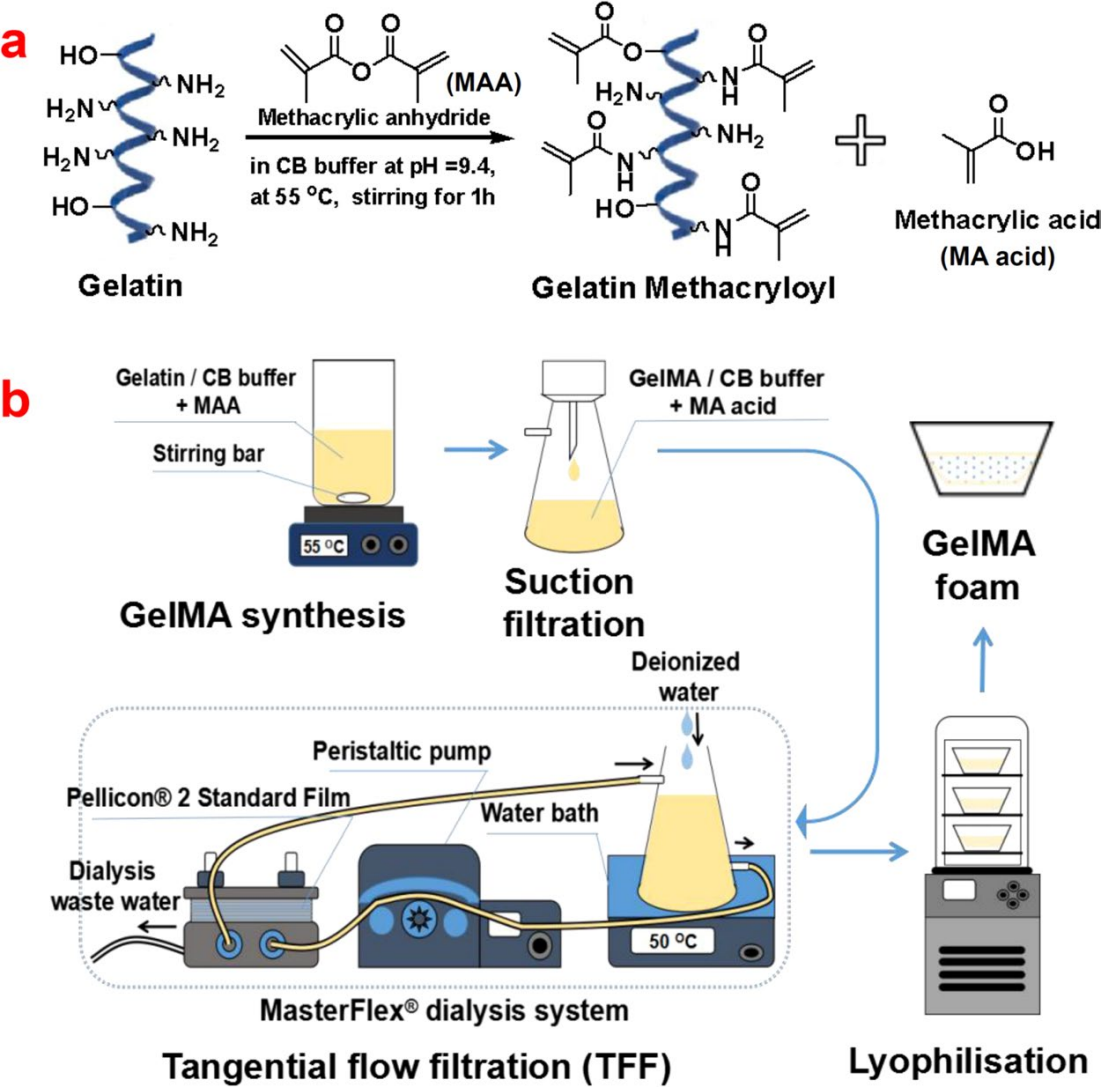
The process of fabrication of target GelMA products. (**a**) Synthesis scheme: two GelMA samples with five batches were prepared at two different molar feed ratios of methacrylic anhydride (MAA) to gelatin (1.859:1 for target DS = 100%, 0.628:1 for target DS = 60%) in carbonate-bicarbonate (CB) buffer system via a one-pot method. (**b**) The process of GelMA production including synthesis, paper filtration, tangential flow filtration (TFF), and lyophilization.

GelMA samples with five different batches were synthesized in the CB buffer system by a one-pot method as illustrated in Fig. 1. Modified synthesis parameters (10 (w/v)% gelatin, 0.25 M CB buffer, a reaction time of 1 h, and reaction temperature of 55 °C) were utilized according to the literature as seen in Table 1^20^. In this study, two types of GelMA samples (target degrees of substitution (DS): DS = 100% and 60%) with five batches were synthesized with feeding mole ratios of MAA to amino groups of gelatin at 1.859:1 and 0.628:1, respectively. GelMA samples (DS = 100% and 60%) with different batches were labeled as DS100_1~5 and DS60_1~5. The obtained products appeared white yellowish. The yields of all GelMA products were around 90% (92%, 93%, 88%, 90%, and 88% for DS100_1~5 and 92%, 94%, 92%, 91%, and 92% for DS60_1~5, respectively), indicating that the current one-pot GelMA batch process can produce consistent yields.

**Table 1:** Reaction parameters of the GelMA synthesis via the one-pot method.

Target samples	Type B gelatin (g)	MAA (mL)	^a^Molar ratio	^b^CB buffer (mL)	^c^T (°C)	^d^t (h)	Initial pH	Yield (%)
DS100_1	10	0.938	1.859	100	55	1	9.4	92
DS100_2	10	0.938	1.859	100	55	1	9.4	93
DS100_3	10	0.938	1.859	100	55	1	9.4	88
DS100_4	10	0.938	1.859	100	55	1	9.4	90
DS100_5	10	0.938	1.859	100	55	1	9.4	88
DS60_1	10	0.317	0.628	100	55	1	9.4	92
DS60_2	10	0.317	0.628	100	55	1	9.4	94
DS60_3	10	0.317	0.628	100	55	1	9.4	92
DS60_4	10	0.317	0.628	100	55	1	9.4	91
DS60_5	10	0.317	0.628	100	55	1	9.4	92

Five batches of GelMA samples with target DS = 100% and target DS = 60% were prepared and denoted as DS100_1~5 and DS60_1~5, respectively.

The symbol of ^a^indicates a molar ratio of methacrylic anhydride (MAA) to amino groups of type B gelatin, ^b^indicates the volume of 0.25 M carbon-bicarbonate (CB) buffer in the reaction system, ^c^indicates the reaction temperature, and ^d^indicates the reaction time. MAA stands for methacrylic anhydride and CB stands for carbonate-bicarbonate.

The main challenge of GelMA synthesis is to precisely control the DS and properties of GelMA in every batch because less controllable reaction systems can lead to less controllable outcomes of GelMA, as seen in Table S1^15^. There are many parameters involved in the reaction of gelatin and methacrylic anhydride (MAA) such as pH, temperature, reaction time, a gelatin concentration, a buffer system, a mole ratio of gelatin and MAA, and stirring speed. The crucial thing of GelMA synthesis is to maintain the pH of the reaction solution since the byproduct (methacrylic acid, MA) can decrease the pH of the solution during the reaction, hindering the forward reaction owing to the protonation of free amino groups. To this end, sequential or dropwise addition of MAA was employed to favor the forward reaction while the pH of the solution was adjusted simultaneously^21,22,25^. However, this method demands and depends on additional labor, which may be less controllable. Recently, Sewald *et al*. reported gelatin type A and type B methacryloyl with various degrees of substitution (DS) using a reaction system of PBS and pH adjustment. Even though they successfully prepared three batches of GelMA with various DS, GelMA materials exhibited relative high standard deviations in terms of methacryloylation and swelling/mechanical properties^24^.

Recent studies reported that a carbonate-bicarbonate (0.25 M CB) buffer could be superior to phosphate buffer saline (0.01 M PBS) in terms of rendering free amino groups reactive via deprotonation and buffering capacity^20,22^. In this respect, a one-pot reaction strategy using the CB buffer at around pH 9 (above the isoelectric point of gelatin) could be ideal, and easy to control the reaction parameters^20^. On the other hand, the CB buffer at even higher pH (pH 11 and 12) can degrade quickly MAA as well as the formed methacrylate groups through hydrolysis, which is not so effective for GelMA synthesis^26^. The buffer capacity of the CB buffer was found to be optimal at around 0.25 M. Another important thing of GelMA synthesis is to improve the miscibility of two reactants (gelatin and MAA) because gelatin is soluble in warm water whereas MAA is insoluble in water. A high concentration of gelatin (above 10%) and a stirring rate of above 500 rpm can be conducive to the homogeneous mixing and reaction of gelatin and MAA because amphiphilic gelatin can serve also as a surfactant, and the high stirring speed may enlarge the reaction interface of gelatin and MAA via stabilizing MAA dispersion in the gelatin solution, subsequently leading to production of homogeneously reacted GelMA. Reaction temperature is also important to completely dissolve gelatin; a temperature between 30–60 °C is acceptable but a temperature above 60 °C might accelerate the backbone degradation of gelatin. A temperature at around 55 °C helps to rapidly dissolve gelatin. That is why the reaction temperature (55 °C) was chosen in this synthesis system. The reaction between gelatin and MAA normally can be complete within 1 hour^20^. Therefore, our one-pot method employing reaction parameters (10 (w/v)% gelatin, 0.25 M CB buffer, a reaction time of 1 h, reaction temperature of 55 °C, an initial pH of 9.4, and a reaction rate of 500 rpm) is exceptionally easy to control the parameters in every batch, resulting in good quality control of GelMA production. In addition, in our system, tangential flow filtration can reduce the dialysis time from several days to several hours through effectively removing the impurities of methacrylic acid and methacrylic anhydride.

### Consistency of the degree of substitution of target GelMA batches (DS100_1~5 and DS60_1~5)

In GelMA production, reproducible methacryloyl functionalization of GelMA is a crucial factor for GelMA with different batches to display consistent hydrogel properties such as swelling behavior, mechanical properties, and degradation after photopolymerization. The amount of methacryloyl groups (AM) in GelMA was quantified by ^1^H-NMR, TNBS, and Fe(III)-hydroxamic acid-based assays (AM_NMR_, AM_TNBS_, and AM_Fe(III)_, respectively). Basically, ^1^H-NMR spectroscopy using TMSP as an internal reference can offer the quantification of both methacrylamide and methacrylate groups in GelMA simultaneously, whereas two different colorimetric methods (TNBS and Fe(III)-hydroxamic assays) provide the quantification of methacrylamide and methacrylate groups in GelMA, respectively^25–27^. The amount of methacryloyl groups (mmole g^−1^) in GelMA can be converted to the degree of substitution (DS; %) through normalization to the amount of the free amino group of original gelatin. Thus, AM_NMR_ amounts to DS_NMR_ whereas the sum of AM_TNBS_ and AM_Fe(III)_ leads to DS_color_.

**Figure 2:**
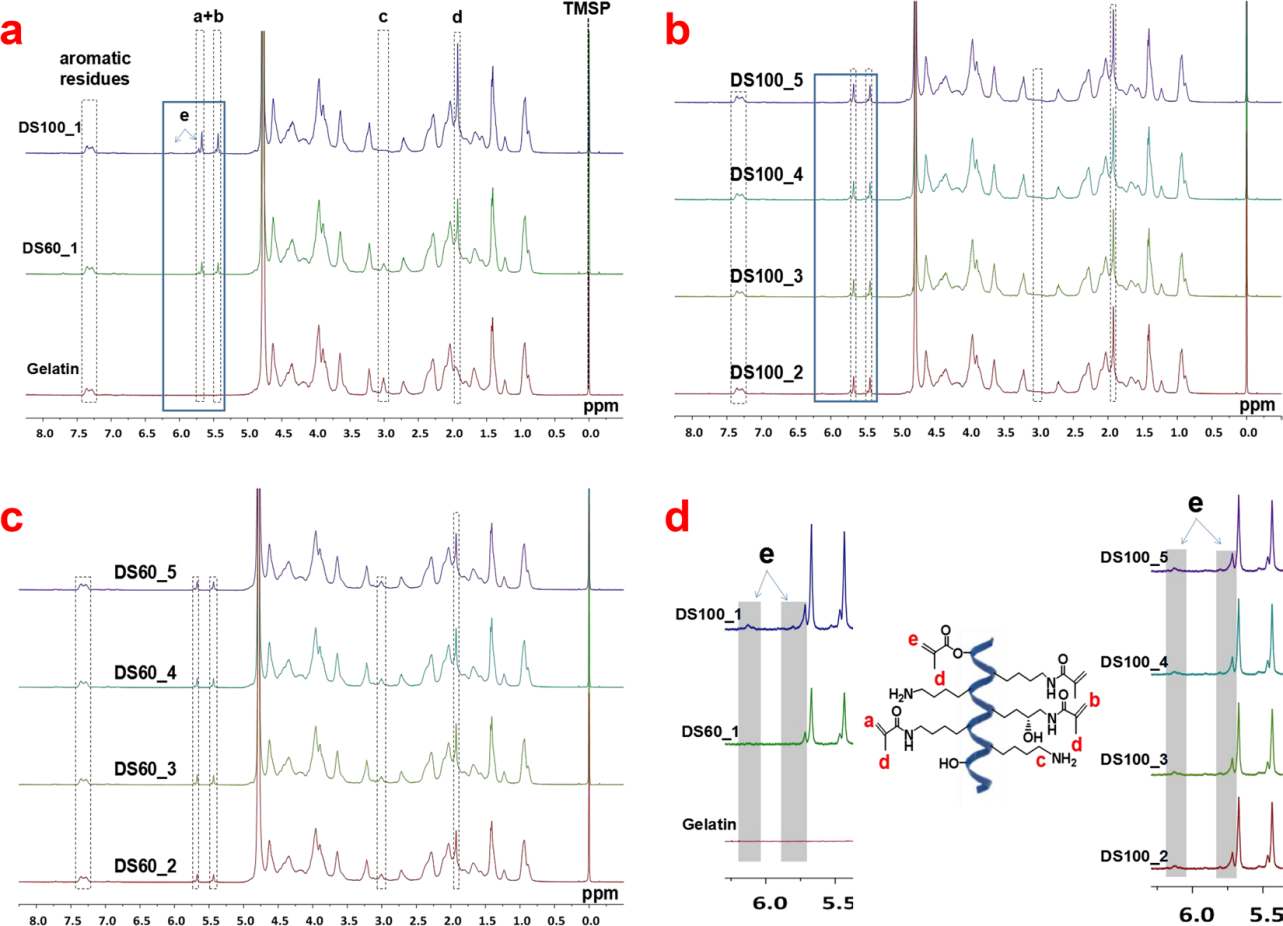
^1^H-NMR spectra of gelatin and two target GelMA samples with five batches (DS100_1~5 and DS60_1~5) in D_2_O. (**a**) ^1^H-NMR spectra of gelatin, DS100_1, and DS60_1. Specific protons of gelatin and GelMA were highlighted as follows: a–e were ascribed to acrylic protons of methacrylamide groups in lysine residues, acrylic protons of methacrylamide groups in hydroxylysine residues, methylene protons of non-modified lysine, methyl protons of methacryloyl groups, and acrylic protons of methacrylate groups, respectively. (**b**) ^1^H-NMR spectra of GelMA (DS100_2~5). (**c**) ^1^H NMR spectra of GelMA (DS60_2~5). (**d**) Zoomed ^1^H-NMR spectra of gelatin and GelMA from 6.40 to 5.30 ppm.

^1^H-NMR spectra were used for determining the amount of methacrylate and methacrylamide groups in GelMA products, as well as for identifying the presence of the byproduct (methacrylic acid) as presented in Fig. 2. In comparison with the ^1^H-NMR spectra of gelatin (Fig. 2a,d), new proton peaks belonging to methacryloyl groups of GelMA appeared between 6.1–5.4 ppm and at 1.9 ppm, and apparently the free lysine signal (NH_2_C**H**_**2**_CH_2_CH_2_CH_2_-) of the unmodified gelatin at 3.0 ppm decreased markedly in DS60_1 and DS100_1 samples. DS100_1 displayed specific chemical shifts between 5.7–5.6 and 5.5–5.4 ppm for acrylic protons (C**H**_**2**_=C(CH_3_)CONH-) of methacrylamide groups and at 1.9 ppm for methyl protons (CH_2_=C(C**H**_**3**_)CO-) of methacryloyl groups, as well as additional small peaks at 6.1 and 5.7 ppm for acrylic protons (C**H**_2_=C(CH_3_)COO-) of methacrylate groups, whereas DS60_1 appeared to show only some specific peaks at about 5.7, 5.5, and 1.9 ppm ascribing to methacrylamide groups (C**H**_**2**_=C(C**H**_**3**_)CONH-) in GelMA. Also, DS100_1 showed a higher peak intensity at 5.7, 5.5, and 1.9 ppm compared to DS60_1. In the ^1^H-NMR spectra, GelMA samples (DS100_1~5 and DS60_1~5) with five different batches showed almost no batch-to-batch difference in terms of methacryloyl functionalization, as seen in Fig. 2b,c. Additionally, all the spectra demonstrated that in all GelMA products there remained little methacrylic acid (the byproduct) whose specific peaks normally appear at 5.7, 5.3 and 1.8 ppm.

**Figure 3:**
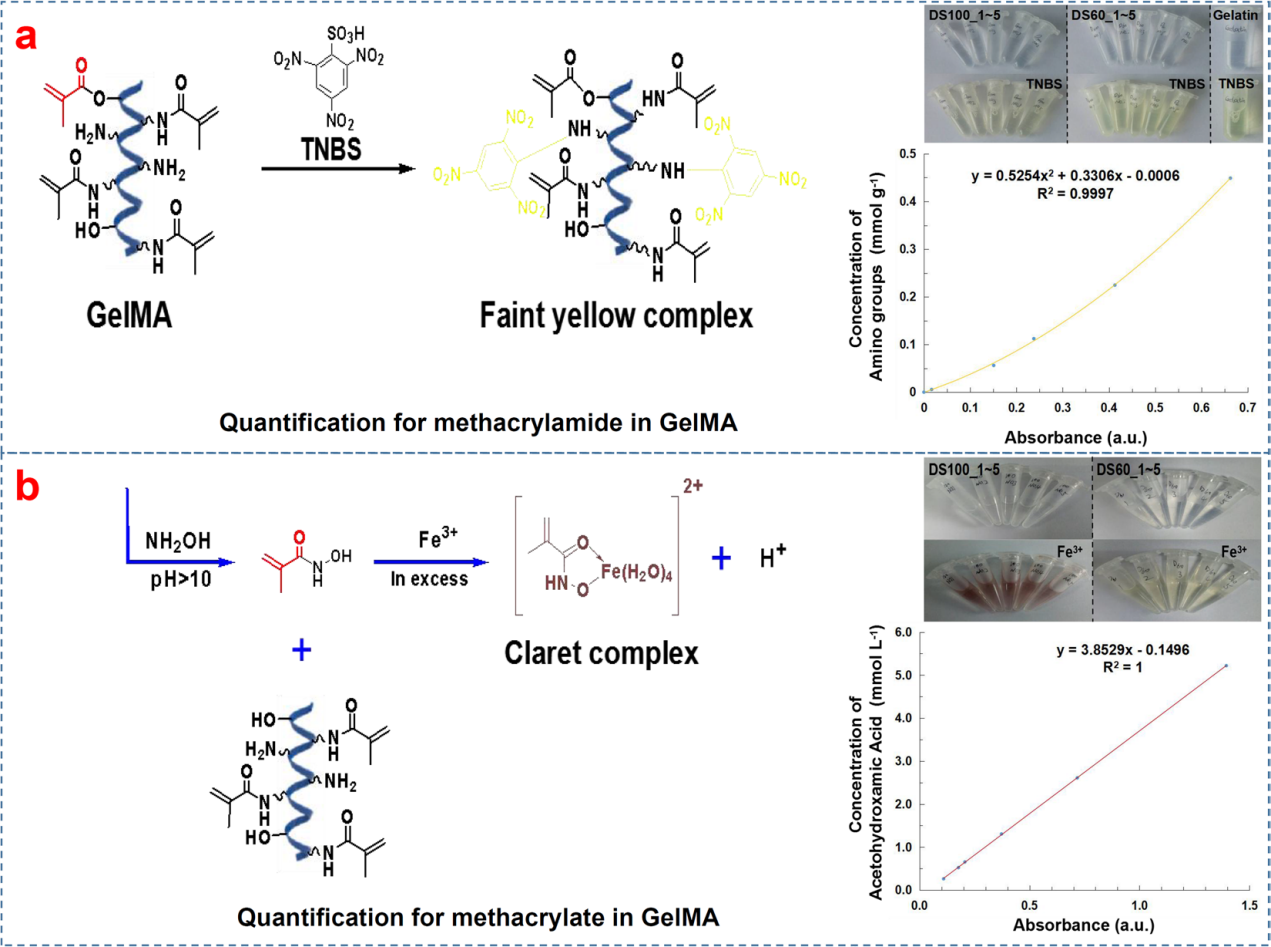
Quantification of the amount of methacryloyl (AM_TNBS_; AM_Fe(III)_) in GelMA by TNBS and Fe(III)-hydroxamic acid-based assays. (**a**) Quantification of methacrylamide in GelMA. The reaction of trinitrobenzenesulfonic acid (TNBS) with primary amines on gelatin and GelMA generated yellow-colored GelMA solutions. The alanine standard curve was used to calculate the amount of free amino groups in gelatin and GelMA. (**b**) Quantification of methacrylate in GelMA. Hydroxylamine reacted with methacrylate groups in GelMA to generate N-hydroxymethacrylamide, which formed a Claret (brown-red) complex with Fe(III) ion. The concentration of methacrylate groups in GelMA was calculated with a standard curve made from a series of standard Fe(III)-acetohydroxamic acid complex solutions. DS60_1~5 displayed no noticeable formation of a brown-red complex owing to no methacrylate groups.

Quantitative results as to the amount of methacryloyl groups (methacrylate and methacrylamide: AM) in DS100_1~5 and DS60_1~5 are summarized in Table 2. The amount of methacryloyl (AM) of DS100_1~5 was recorded by NMR, TNBS, and Fe(III) assays, whereas that of DS60_1~5 was recorded by NMR and TNBS methods since methacrylate groups in DS60_1~5 were not detected by colorimetric Fe(III)-based assay, as displayed in Fig. 3a,b. This was because, in lowly substituted GelMA (DS60_1~5), methacrylic anhydride could dominantly react with free amino groups of lysine and hydroxylysine, resulting in the formation of methacrylamide. The AM_NMR_ value of each GelMA was similar to the sum of AM_TNBS_ and AM_Fe(III)_ of each GelMA. Each GelMA group showed a similar degree of substitution (DS; the methacryloyl functionalization). DS100_1~5 samples exhibited DS_color_ values of 102.29 ± 0.38, 101.32 ± 0.31, 102.54 ± 0.94, 101.31 ± 0.13, 102.83 ± 1.10, respectively (n = 3, one-way ANOVA, p = 0.139). Lowly substituted GelMA, DS60_1~5, had DS_color_ values of 58.62 ± 1.27, 60.84 ± 0.90, 59.54 ± 1.18, 58.50 ± 1.20, 61.47 ± 1.09, respectively (n = 3, one-way ANOVA, p = 0.063). Overall, the results regarding the DS of GelMA (DS100_1~5 and DS60_1~5) demonstrate that the current one-pot batch method for highly and lowly substituted GelMA can produce GelMA with desired DS values and little batch-to-batch variation.

**Table 2:** The amount of methacryloyl (AM, mmole g^−1^) and the degree of substitution (DS, %) of target DS100_1~5 and DS60_1~5.

Target Samples	NMR assays		Colorimetric methods		
	AM_NMR_ (mmole g^−1^)	DS_NMR_ (%)	AM_TNBS_ (mmole g^−1^)	AM_Fe(III)_ (mmole g^−1^)	DS_color_ (%)
DS100_1	0.3266 ± 0.0023	102.61 ± 0.72	0.3141 ± 0.0011	0.0116 ± 0.0001	102.29 ± 0.38
DS100_2	0.3229 ± 0.0019	101.45 ± 0.60	0.3117 ± 0.0009	0.0109 ± 0.0001	101.32 ± 0.31
DS100_3	0.3273 ± 0.0030	102.83 ± 0.94	0.3131 ± 0.0010	0.0134 ± 0.0020	102.54 ± 0.94
DS100_4	0.3232 ± 0.0008	101.54 ± 0.25	0.3111 ± 0.0002	0.0115 ± 0.0002	101.31 ± 0.13
DS100_5	0.3273 ± 0.0030	102.83 ± 0.94	0.3147 ± 0.0016	0.0127 ± 0.0019	102.83 ± 1.10
DS60_1	0.1939 ± 0.0025	60.92 ± 0.79	0.1866 ± 0.0040	\	58.62 ± 1.27
DS60_2	0.1970 ± 0.0011	61.87 ± 0.35	0.1937 ± 0.0029	\	60.84 ± 0.90
DS60_3	0.1964 ± 0.0013	61.70 ± 0.41	0.1896 ± 0.0037	\	59.54 ± 1.18
DS60_4	0.1849 ± 0.0015	58.09 ± 0.47	0.1863 ± 0.0039	\	58.50 ± 1.20
DS60_5	0.1968 ± 0.0014	61.83 ± 0.44	0.1957 ± 0.0034	\	61.47 ± 1.09

AM_NMR_, AM_TNBS_, and AM_Fe(III)_ were measured by ^1^H NMR spectra, TNBS assay, and Fe(III) assay, respectively. The degree of substitution (DS) of GelMA was calculated using either AM_NMR_ or a combination of the colorimetric results (AM_TNBS_ and AM_Fe(III)_): DS_NMR_ = AM_NMR_/0.3184 × 100 (%) and DS_color_ = (AM_TNBS_ + AM_Fe(III)_)/0.3184 × 100 (%). The amount of the free amino groups of gelatin was 0.3184 mmole g^−1^. No methacrylate groups in DS60_1~5 were detected by Fe(III) assay. Data represent means ± standard deviations.

### Consistency of the secondary structure of GelMA batches

**Figure 4:**
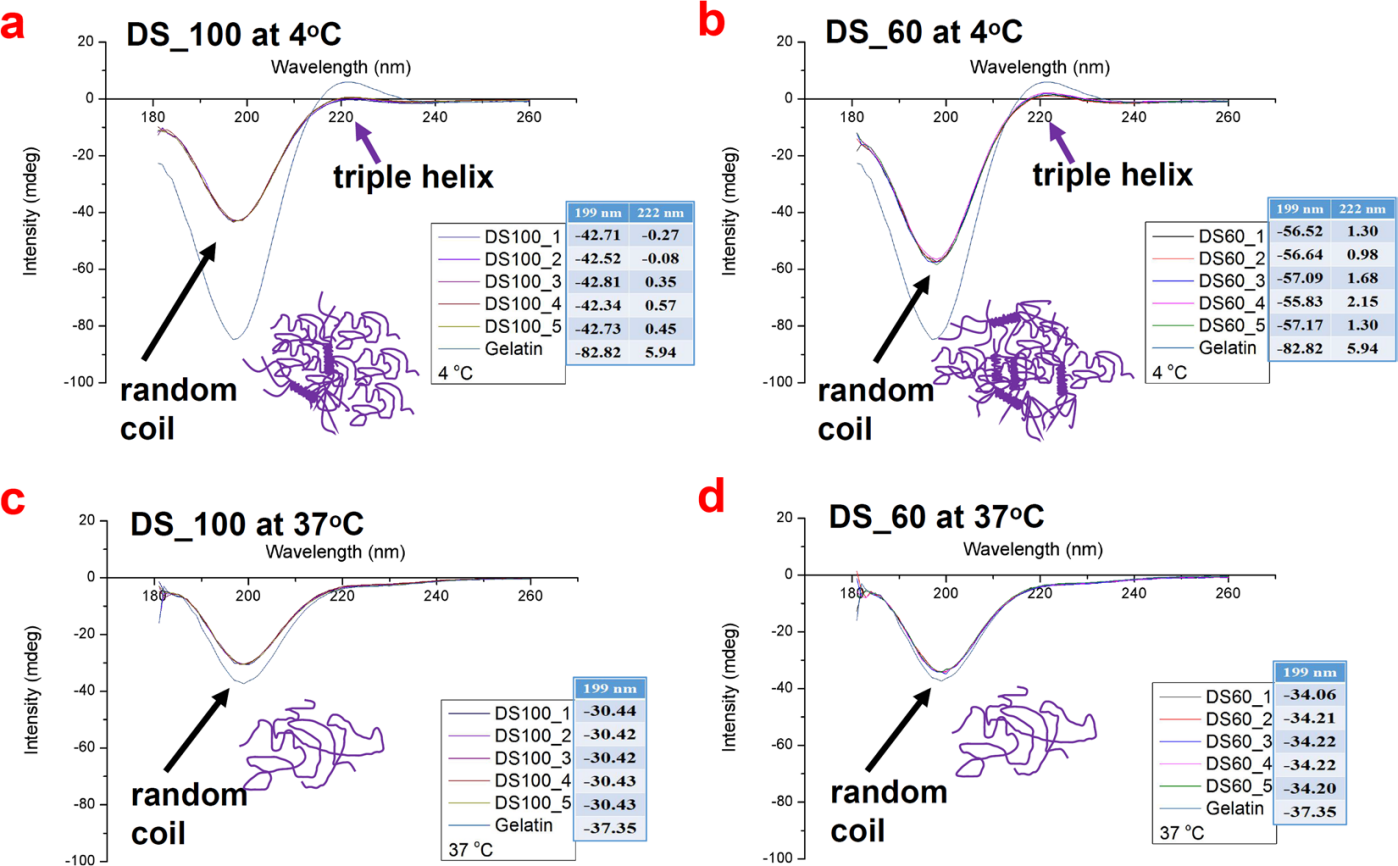
CD spectra of gelatin and GelMA (DS100_1~5 and DS60_1~5) in deionized water at a concentration of 0.2 mg mL^−1^. (**a**) DS100_1~5 at 4 °C. (**b**) DS60_1~5 at 4 °C. (**c**) DS100_1~5 at 37 °C. (**d**) DS60_1~5 at 37 °C. Almost no batch-to-batch variation in CD curves was observed among DS100_1~5 or DS60_1~5, indicating that each gelMA group (DS100_1~5 or DS60_1~5) exhibited its own secondary structure. DS100_1~5 and DS60_1~5 exhibited a marked increase of the random coil portion in the intensity at 199 nm and a slight decrease of the triple helix formation in the intensity at 222 nm at 4 °C, compared with gelatin. An increase in the DS increased the random coil conformation of GelMA and decreased the triple-helix formation of GelMA. However, both DS100 and DS60 samples, like gelatin, lost the triple-helix formation at 37 °C and exhibited a similar amount of the random coil conformation at 222 nm.

Gelatin exhibits partial triple helix formation at a low temperature in aqueous solutions and forms random coil structure upon heating. Its transition from triple helix to random coil is reversible. In comparison with gelatin, GelMA samples (DS100_1~5 and DS60_1~5) were expected to retain a certain degree of the secondary structure of gelatin even though the methacryloyl functionalization of GelMA can potentially interfere with helix formation^23,28^. Figure 4 shows the CD spectra of gelatin, highly substituted GelMA (DS100_1~5), and lowly substituted GelMA (DS60_1~5) that provide the information of their secondary structure at 4 °C and 37 °C. As presented in Fig. 4a,b, DS100_1~5 and DS60_1~5 displayed similarly a distinct rise in the intensity at 199 nm at 4 °C, compared with gelatin. The intensity of highly substituted GelMA (DS100_1~5) at 199 nm at 4 °C, ascribing to a portion of random coil formation, was slightly higher than that of lowly substituted GelMA (DS60_1~5), suggesting that higher methacryloyl functionalization of GelMA could further elicit random coil formation. On the other hand, the triple-helix contents of GelMA samples (DS100_1~5 and DS60_1~5) at 222 nm at 4 °C decreased markedly, compared with gelatin. DS100_1~5 exhibited a slightly lower intensity at 222 nm than DS60_1~5, indicating that lowly substituted GelMA could retain a more amount of the triple-helix formation at 4 °C than highly substituted GelMA. Additionally, GelMA with a higher DS (DS100_1) exhibited a less temperature-sensitive phase transition (helix-random coil transition) compared with GelMA with a lower DS (DS60_1), as seen in Fig. S1. It is speculated that the methacryloyation of free amino groups or hydroxyl groups in gelatin chains could reduce interchain or intrachain hydrogen bonding in the triple helix, leading to an increase in the random coil portion and a decrease in the triple helix formation^23^. Glycine-Proline-hydroxyproline tripeptides have been found to participate in the triple helix formation^29–31^. Hydroxyl groups of hydroxyproline can react with methacrylic anhydride (MAA) especially in a high feed of MAA^25,26^. Highly substituted GelMA (DS100_1~5) possessed the methacrylate group of around 0.01 mmole g^−1^ most likely from the reaction of hydroxyproline and MAA, which is presumed to obstruct partially the triple-helix formation. On the other hand, all GelMA as well as gelatin showed similar patterns in the CD spectra at 37 °C and exhibited a large increase in the intensity at 199 nm relative to the samples at 4 °C, as seen in Fig. 4c,d, indicating that GelMA materials including gelatin experience a helix-coil transition on heating. Highly substituted GelMA (DS100_1~5) showed a slightly higher intensity at 199 nm than lowly substituted GelMA (DS60_1~5). The triple-helix contents of all GelMA and gelatin at 222 nm at 37 °C decreased significantly compared with those at 4 °C, indicating that GelMA samples (DS100_1~5 and DS60_1~5) as well as gelatin appeared to completely lose the triple-helix formation and behaved like random coils at 37 °C. In addition, the CD spectra patterns of each GelMA group (DS100_1~5 or DS60_1~5) were almost the same even at different temperatures, meaning that each GelMA group with five batches was consistent in the secondary structure formation. GelMA (DS100_1~5 and DS60_1~5) displayed a higher degree of consistency not only in the composition of methacryloyl, but also in the protein secondary structure.

### Consistency of hydrogel properties of GelMA batches (DS100_1~5 and DS60_1~5)

Gelatin undergoes only physical gelation at a low temperature whereas GelMA can form a physical gel at a low temperature and additionally form a chemical hydrogel via photopolymerization owing to photosensitive methacryloyl functionalization. GelMA hydrogels exhibit tailorable swelling behavior and mechanical properties, which depend on mainly their degree of substitution, their concentration and selected curing parameters (light intensity, exposure time of irradiation, and amounts of an initiator). Here, the batch-to-batch variation of hydrogel properties of GelMA (DS100_1~5 and DS60_1~5) was investigated in terms of swelling and mechanical stiffness.

**Figure 5:**
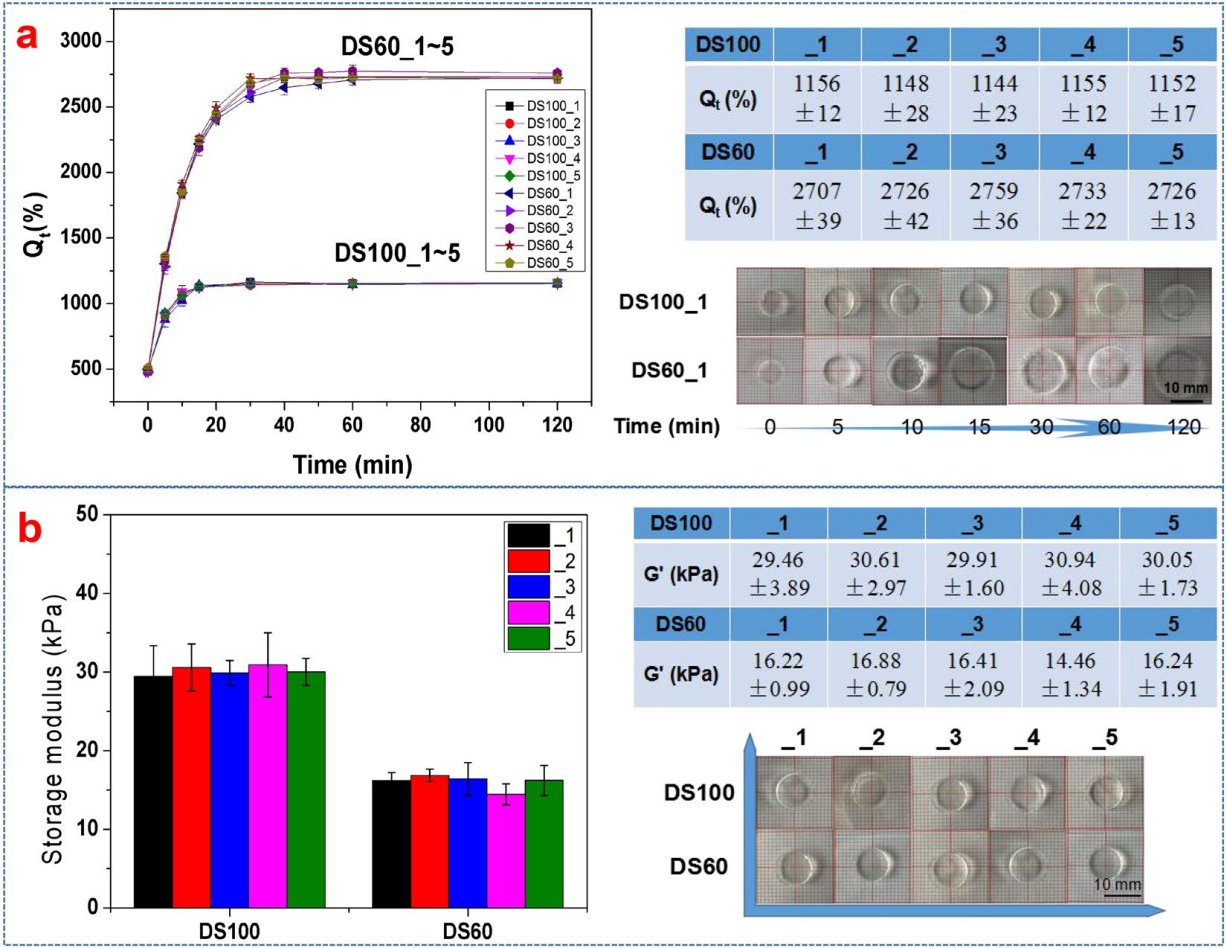
Consistency of swelling and mechanical properties of GelMA (DS100_1~5 and DS60_1~5) hydrogels. (**a**) Swelling behavior of DS100_1~5 and DS60_1~5 at 20 (w/v)%. Swelling behavior of each hydrogel group was consistent. Highly substituted GelMA (DS100_1~5) hydrogels exhibited 2.5-fold less swelling than lowly substituted GelMA (DS60_1~5) hydrogels. (**b**) Mechanical properties of GelMA hydrogels. Storage moduli of GelMA (DS100_1~5 and DS60_1~5) hydrogels at 20 (w/v)% were measured at 0.1% strain and 0.1–10 Hz at 37 °C. D100_1~5 and DS60_1~5 hydrogels exhibited storage moduli of around 30 kPa and 16 kPa, respectively.

First, GelMA hydrogels were fabricated through a simple method: Each 20 (w/v)% GelMA solution containing 0.5 (w/v)% I2959 was placed in a mold (8 mm in diameter and 1 mm in thickness) and then cured by 365 nm UV light (3.5 mW cm^−2^ and 5 minutes). As shown in Fig. 5a, GelMA (DS100_1~5 and DS60_1~5) bulk hydrogels exhibited structural integrity after photo-crosslinking. When GelMA hydrogels were soaked in DI water at an elevated temperature (50 °C) to expedite the swelling process, they began to swell and reached a swelling equilibrium within 60 min. In addition, any degradation of DS100 and DS60 hydrogels was not observed at the elevated temperature during the swelling test. DS100_1~5 hydrogels exhibited a lower swelling degree (%) compared with DS60_1~5 hydrogels. Swelling degrees of DS100_1~5 hydrogels were 1156 ± 12, 1148 ± 26, 1144 ± 23, 1155 ± 12, and 1152 ± 17%, respectively (n = 3, one-way ANOVA, p = 0.489) whereas those of DS60_1~5 were 2707 ± 39, 2726 ± 42, 2759 ± 36, 2733 ± 22, and 2726 ± 13%, respectively (n = 3, one-way ANOVA, p = 0.078). DS100_1~5 hydrogels should have a higher crosslinking density and a smaller mesh size compared to DS60_1~5, owing to a higher degree of methacryloyl functionalization, subsequently leading to less swelling. Additionally, the results demonstrated that each GelMA hydrogel group (DS100_1~5 or DS60_1~5) showed a higher degree of consistency in swelling behavior. The swelling of hydrogels is an important feature for the diffusion behavior of small molecules (nutrients and waste) in cell culture and drug delivery systems. Consistent swelling behavior of GelMA (DS100_1~5 or DS60_1~5) hydrogels could be used as a predictable basic reference for various bioapplications.

As to mechanical properties of GelMA (DS100_1~5 and DS60_1~5) hydrogels at 20 (w/v)%, highly substituted GelMA materials (DS100_1~5) with an average of 30.20 ± 0.57 kPa were 1.9-fold stiffer than lowly substituted GelMA (DS60_1~5) with an average of 16.04 ± 0.93 kPa. As seen in Fig. 5b, GelMA (DS100_1~5 and DS60_1~5) hydrogels exhibited little batch-to-batch variance in their mechanical properties. Storage moduli of DS100_1~5 were 29.46 ± 3.89, 30.61 ± 2.97, 29.91 ± 1.60, 30.94 ± 4.08, and 30.05 ± 1.73 kPa (n = 3, one-way ANOVA, p = 0.975) whereas those of DS60_1~5 were 16.22 ± 0.99, 16.88 ± 0.79, 16.41 ± 2.09, 14.46 ± 1.34, and 16.24 ± 1.91 (n = 3, one-way ANOVA, p = 0.398). Recently, Seward *et al*. reported mechanical properties of GelMA with various degrees of methacrylation (0.3~1.0 mmole g^−1^), and the storage modulus of each GelMA showed relatively high standard deviations potentially owing to a high batch-to-batch variance^24^. Even mechanical properties of lowly methacrylated GelMA with the methacrylation of around 0.3 mmole g^−1^ were not statistically different from those of highly methacrylated GelMA with the methacrylation of around 0.6 mmole g^−1^, assumingly because of the much influence of the physical crosslinking of lowly methacrylated GelMA. In our case, the mechanical and swelling properties of GelMA hydrogels showed a direct correlation with the degree of methacrylation. It is potentially because the hydrogels were prepared above 37 °C, which could rule out the physical gelation. The chemical gelation and crosslinking density of GelMA hydrogels formed by light could be a dominant factor of determining their mechanical properties and swelling behavior, resulting in distinct mechanical properties of each GelMA group and a low batch-to-batch difference within each GelMA group. GelMA hydrogels with a high degree of consistency and tailorability in mechanical properties could be a highly versatile tool for tissue engineering applications since tunable mechanical properties of soft hydrogels have been used to regulate cellular behavior such as proliferation, migration, and differentiation^32^.

### Consistency of biodegradability and cell viability of GelMA (DS100_1~5 and DS 60_1~5) hydrogels

**Figure 6:**
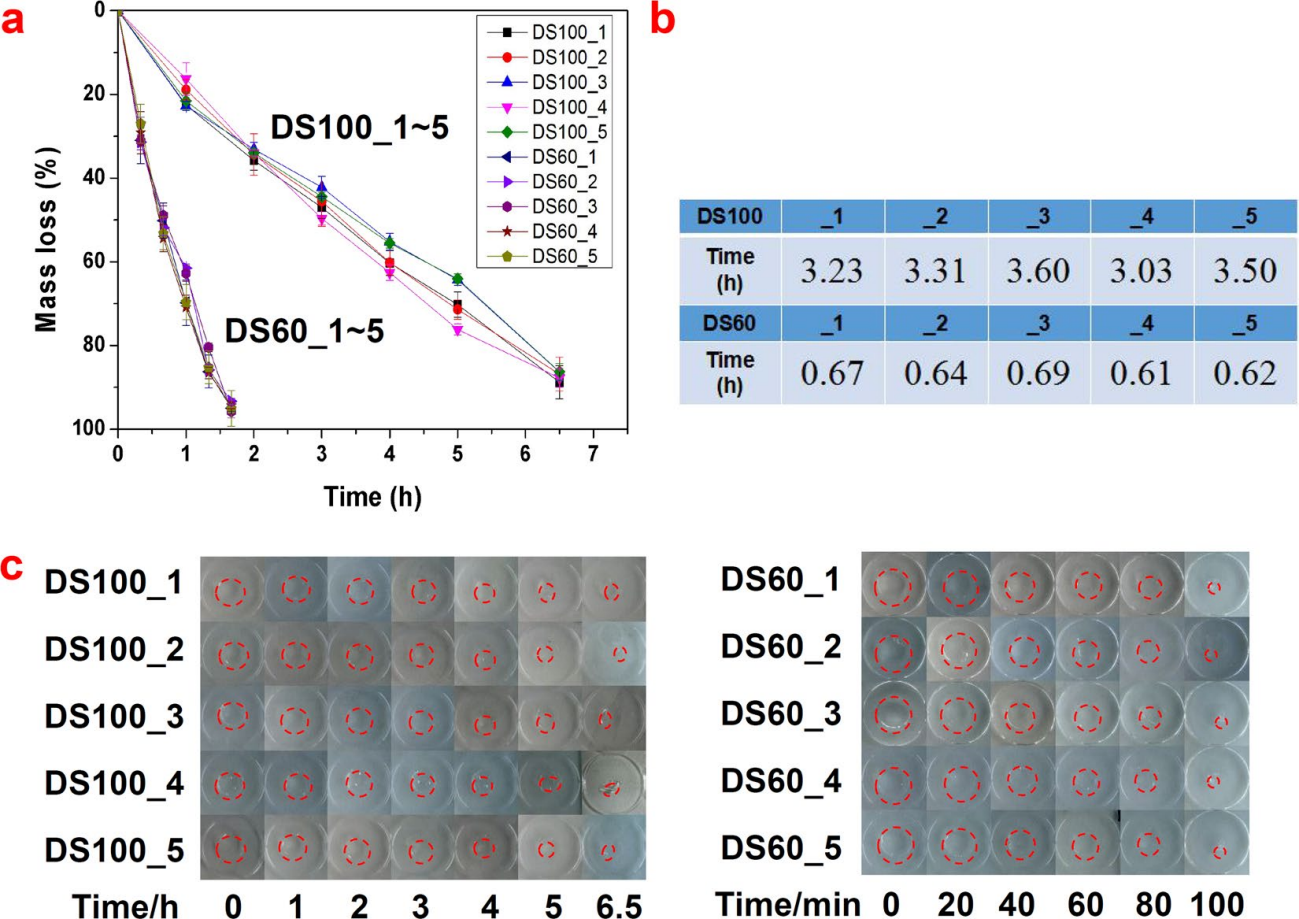
Consistency of degradation properties of GelMA hydrogels (DS100_1~5 and DS60_1~5). (**a**) Mass loss profiles of 20 (w/v)% GelMA hydrogels under an accelerated enzymatic condition. GelMA hydrogels experienced fast enzymatic degradation at a collagenase concentration of 0.5 mg mL^−1^ (125 CDU mg^−1^) in Hank’s Balanced Salt Solution containing 3 mM CaCl_2_ at 37 °C. Data represents means ± standard deviations (n = 3). (**b**) Half-life time required for the mass of GelMA to reach half its original mass. (**c**) Optical images of enzymatic degradation of GelMA hydrogels. The outline of GelMA hydrogels was drawn in red. DS100_1~5 hydrogels degraded much more slowly than DS60_1~5.

Biodegradability has gained considerable attention in drug delivery and tissue engineering applications as a desirable feature of hydrogel materials. GelMA hydrogels exhibit enzymatic degradation properties; indeed, GelMA retains enzyme-sensitive sequences (proline-X-glycine-proline-, X: a neutral amino acid) as its parent gelatin and collagen do. Here, accelerated enzymatic degradation tests of GelMA (DS100_1~5 and DS60_1~5) hydrogels were conducted to investigate consistency of their degradation behavior. As displayed in Fig. 6a–c, the enzymatic degradation of bulk GelMA hydrogels appeared apparent, and their degradation speed was highly dependent on the DS of GelMA, which affects dominantly the crosslinking density of GelMA hydrogels. The higher the DS of GelMA hydrogels, the slower their degradation. DS100_1~5 hydrogels under the accelerated degradation conditions lost half of their masses at around 3.33 h, and DS60_1~5 hydrogels did at around 0.65 h. Actual half-lives of DS100_1~5 hydrogels were 3.23, 3.31, 3.60, 3.03, and 3.50 h, respectively whereas those of DS60_1~5 were 0.67, 0.64, 0.69, 0.61, and 0.62 h, respectively. Each GelMA hydrogel group followed a similar degradation pattern, which showed that there seemed to be little batch-to-batch difference in hydrogel structure and susceptibility to enzyme degradation within each hydrogel group. We presume that the crosslinking density caused by photocrosslinking of the methacryloyl group could be the main factor of making a difference in the degradation rate of GelMA (DS100_1~5 and DS60_1~5) hydrogels. GelMA DS100_1~5 hydrogels with a higher DS can slow down the enzymatic degradation owing to a higher crosslinking density in the hydrogels, compared with GelMA DS60_1~5 hydrogels with a lower DS^13^.

**Figure 7:**
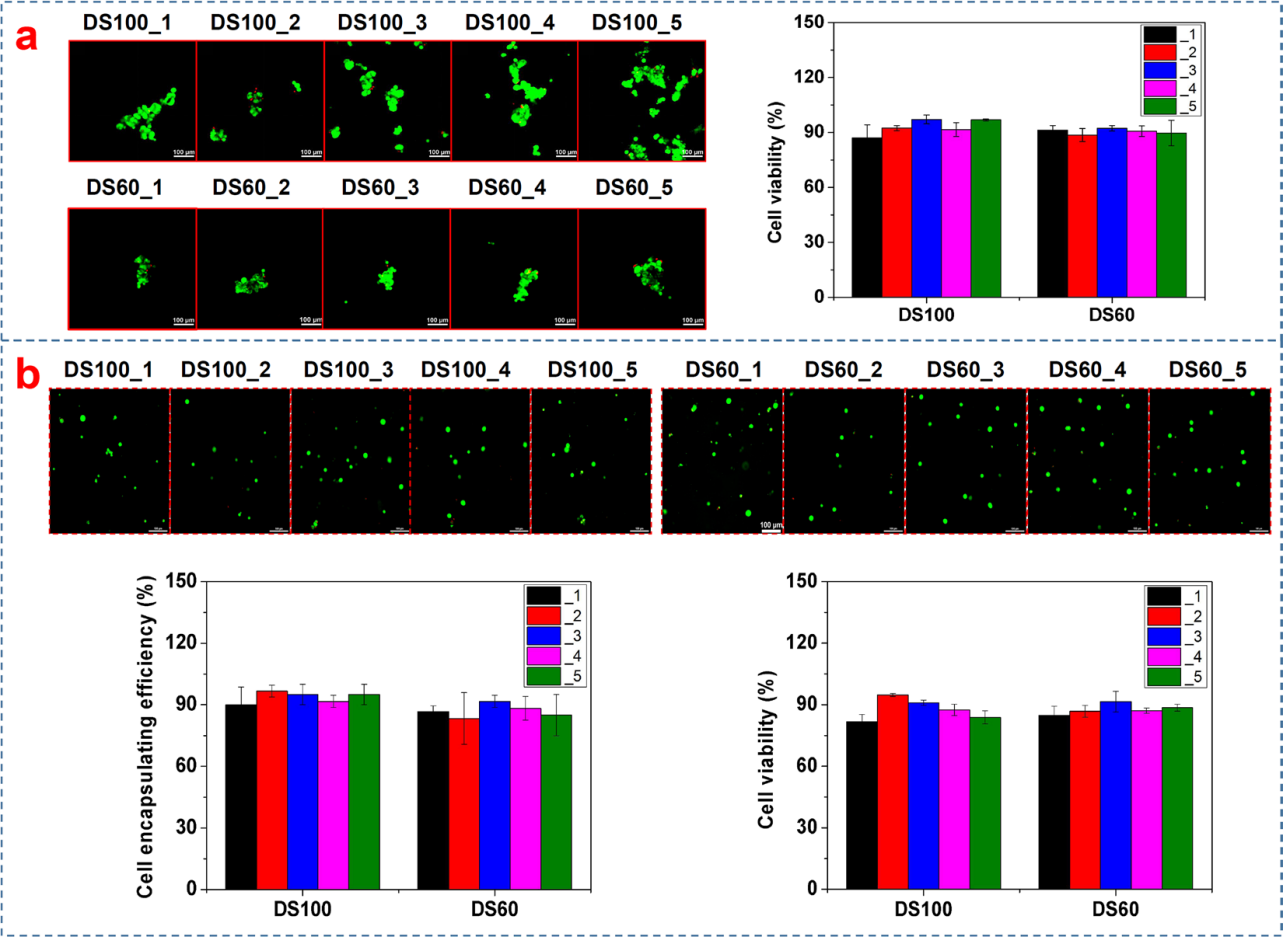
Cell viability of GelMA (DS100_1~5 and DS60_1~5) hydrogels. (**a**) Cell viability of Huh7.5 cells cultured on GelMA hydrogels (DS100_1~5 and DS60_1~5). All samples showed cell viability of above 87%. GelMA hydrogels exhibited no significant difference in cell viability among the samples (n = 3, one-way ANOVA, p > 0.05) The live/dead images of Huh7.5 cells on GelMA hydrogels. Cells on GelMA hydrogels seemed to form cell clusters. (**b**) Encapsulation efficiency and cell viability of Huh7.5 cells inside GelMA hydrogels. Both DS100_1~5 and DS60_1~5 hydrogels exhibited good cell encapsulation efficiency and cell viability. Cells inside GelMA hydrogels displayed a round shape compared to those on GelMA hydrogels. Green and red colors indicate live and dead cells, respectively. Scale bar = 100 μm.

Also, GelMA, like its parents (gelatin and collagen), maintains cell binding sites (e.g. RGD). Cell binding affinity of GelMA is an important feature that can promote cell viability and affect cell behavior such as cell proliferation and differentiation. We investigated cell viability of Huh7.5 cells cultured on and inside GelMA hydrogels (DS100_1~5 and DS60_1~5). As presented in Fig. 7a, GelMA hydrogels (DS100_1~5 and DS60_1~5) exhibited cell viability of above 87%. Cell viability of DS100_1~5 and DS60_1~5 hydrogels was not significantly different from one another (one-way ANOVA, p = 0.812). Cell viability values of DS100_1~5 hydrogels were 87.1 ± 7.1%, 92.4 ± 1.4%, 97.1 ± 2.4%, 91.6 ± 3.8%, and 96.9 ± 0.5%, respectively whereas those of DS60_1~5 hydrogels were 91.3 ± 2.5%, 88.6 ± 3.6%, 92.3 ± 1.5%, 90.7 ± 2.9%, and 89.7 ± 7.0%, respectively. Cells on GelMA (DS100_1~5 and DS60_1~5) hydrogels appeared as cell clusters possibly because GelMA substrates were soft and compliant. Huh 7.5 cells on soft hydrogels tended to form cell clusters or spheroids^13^. Cell clusters on DS100_1~5 hydrogels looked slightly more scattered than those on DS60_1~5. It was speculated that relatively stiffer DS100_1~5 hydrogel substrates could allow cells to be more spread and scattered, as compared to DS60_1~5 hydrogel substrates.

In addition, cells were encapsulated inside GelMA hydrogels as presented in Fig. 2b. The average (93.7 ± 5.2%) of cell encapsulation efficiency of DS100_1~5 hydrogels was slightly higher than that (87.0 ± 7.3%) of DS60_1~5. The cell encapsulation efficiency of each GelMA group showed no statistical difference (one-way ANOVA, p > 0.05). Cells encapsulated inside DS100_1~5 hydrogels displayed an average cell viability of 87.7 ± 3.7% whereas those inside DS60_1~5 had an average cell viability of 87.8 ± 5.3%. In comparison to cells grown on GelMA hydrogels, cells inside GelMA hydrogels exhibited a round morphology possibly owing to the fact that cells were surrounded and packed by dense hydrogel matrices in a three-dimensional manner. Overall both DS100_1~5 and DS60_1~5 hydrogels were found to offer good cell viability with good batch-to-batch consistency.

In this report, we prepared two kinds of target GelMA (DS100_1~5 and DS60_1~5) with five batches using the one-pot synthesis method. GelMA (highly and lowly substituted versions: DS100_1~5 and DS60_1~5) batches displayed high reproducibility and controllability in their photocurable functionalization, protein secondary structures, mechanical properties, degradation behavior, and cell viability. GelMA with almost no batch-to-batch difference in structural and functional properties could be used as a highly versatile source for bioapplications that require a high degree of consistency in properties and performances. In addition, GelMA one-pot synthesis and characterization methods described in this work could be a useful guideline for quality control of GelMA production and further could be extended to the methacryloyl functionalization of other biopolymers with amino and hydroxyl functional groups for their bioapplications.

## Methods

### Materials

Gelatin (type B, 250 bloom), sodium carbonate, sodium bicarbonate decahydrate, sodium hydroxide, acetohydroxamic acid, hydroxylamine, sodium dodecyl sulfate, and alanine were purchased from Aladdin (Shanghai, China). Methacrylic anhydride (MAA), iron(III) perchloride, 2-hydroxy-4′-(2-hydroxyethoxy)-2-methylpropiophenone (I2959), collagenase (IA, 125 CDU/mg), and 2,4,6-trinitrobenzene sulfonic acid (TNBS) were purchased from Sigma-Aldrich (Shanghai, China). Deuterium oxide (D_2_O) and 2,2,3,3-D_4_ (D, 98%) sodium-3-trimethylsilylpropionate (TMSP) were obtained from Cambridge Isotope Laboratories (Andover, USA). Hanks’ Balanced Salt Solution (HBSS) (10X), Dulbecco’s Modified Eagle’s Medium, fetal bovine serum, penicillin/streptomycin, and Live/Dead® Cell Viability/Cytotoxicity kit were purchased from Life Technologies (Shanghai, China). All the reagents were used as received.

### Preparation of gelatin methacryloyl (GelMA, target DS = 100 and 60%) with five batches

Two kinds of gelatin methacryloyl (GelMA, target DS = 100 and 60%) materials with five different batches (DS100_1~5 and DS60_1~5) were prepared as illustrated in Fig. 1a,b. Details regarding reaction parameters are presented in Table 1. In brief, type B gelatin (10 g, 250 bloom, 3.18 mmole of free amino groups) was dissolved at 10 (w/v)% in carbonate-bicarbonate (CB) buffer (0.25 M, 100 mL) at 55 °C, and then the pH of the gelatin solutions was adjusted to 9.4. Two different amounts (0.938 mL for target DS = 100% and 0.317 mL for target DS = 60%) of methacrylic anhydride (MAA, 94%) were separately added to the gelatin solutions under magnetic stirring at 500 rpm. The reaction proceeded for 1 h at 55 °C, and the final pH of the reaction solutions was adjusted to 7.4 to stop the reaction. After being filtered, the solutions were dialyzed against water at 50 °C in a MasterFlex^®^ tangential flow filtration (TFF) system equipped with Pellicon^®^ 2 cassette (Darmstadt, Germany) containing a 10 k Da Biomax membrane, and lyophilized to obtain the final solid products. The average yield on GelMA products was around 90%.

### Degree of substitution of target GelMA (DS100_1~5 and DS60_1~5)

For quantification of methacrylamide groups in GelMA, ^1^H-NMR (Avance I 400 MHz, Bruker, Rheinstetten, Germany) spectroscopy and 2,4,6-Trinitrobenzenesulfonic acid (TNBS) assay were employed, whereas ^1^H-NMR and Fe(III)-hydroxamic acid-based assay were harnessed for quantification of methacrylate groups in GelMA^20,25–27^. For ^1^H-NMR spectroscopy of gelatin and GelMA, 40 mg of each sample was dissolved in 800 µL of deuterium oxide with 0.1 w/v% 3-(trimethylsilyl)propionic-2,2,3,3-d_4_ (TMSP) acid as an internal reference, and all ^1^H-NMR-spectra were measured at 40 °C. Before the interpretation, phase corrections were applied to all spectra to obtain purely absorptive peaks, baselines were corrected, and the chemical shift scale was adjusted to the TMSP signal (δ (^1^H) = 0 ppm). The amount of methacryloyl groups (methacrylamide and methacrylate groups; AM_NMR_) in GelMA was calculated from ^1^H-NMR spectroscopy by the following formula:

$$\begin{array}{rcl}{{\rm{AM}}}_{{\rm{NMR}}}({\rm{mmole}}\,{{\rm{g}}}^{-1}) & = & \frac{\int {\rm{methacryloyl}}\,({\rm{the}}\,{\rm{peaks}}\,{\rm{at}}\,\,5.6\mbox{--}5.8\,{\rm{ppm}})}{\int \mathrm{TMSP}\,\,({\rm{at}}\,0\,{\rm{ppm}})}\\  &  & \times \,\frac{9{\rm{H}}}{1{\rm{H}}}\times \frac{{\rm{n}}\,{\rm{mmole}}\,({\rm{TMSP}})}{{\rm{m}}\,{\rm{g}}\,({\rm{GelMA}})};\end{array}$$

Also, TNBS assay was performed to quantify the remaining free amino groups in gelatin and GelMA. GelMA and gelatin samples were separately dissolved at 1.6 mg mL^−1^ in 0.1 M sodium bicarbonate buffer. Then, 0.5 mL of each sample solution was mixed with 0.5 mL of a 0.1% TNBS solution in 0.1 M sodium bicarbonate buffer and then was incubated for 2 h. Next, 0.25 mL of 1 M hydrochloric acid and 0.5 mL of 10 (w/v)% sodium dodecyl sulfate were added to stop the reaction. The absorbance of each sample was measured at 335 nm. An alanine standard curve was used to determine the amino group concentration with standard sample solutions prepared at 0, 0.8, 8, 16, 32, and 64 µg mL^−1^. The amount of free amino groups in 1 g of gelatin is 0.3184 mmol, based on the TNBS result. The amount of methacrylamide groups in GelMA, denoted as AM_TNBS_, was obtained by subtracting the amount of the remaining free amino groups in GelMA from the amount of free amino groups in gelatin.

Fe(III)-hydroxamic acid-based assay was used for quantification of the amount of methacrylate groups in GelMA (AM_Fe(III)_). Briefly, 100 µL of 0.5 M hydroxylamine hydrochloride was combined with 100 µL of 1 M NaOH, and then 200 µL of each GelMA solution (50 mg mL^−1^) was transferred to the combined solution. The mixture was incubated at room temperature for 10 min after being vortexed for 30 s. Then, 550 µL of a 0.5 M iron(III) perchloride solution in 0.5 M HCl was added to the mixture. After the mixture solution was vortexed for 30 s, UV-vis absorption spectra were recorded from 420 to 700 nm in a microplate with 200 µL of the solution. The absorbance at 500 nm was used to quantify AM_Fe(III)_ via utilizing a standard curve prepared with a series of acetohydroxamic acid solutions (5.0 × 10^−3^, 2.5 × 10^−3^, 1.25 × 10^−3^, 6.25 × 10^−4^, 5.0 × 10^−4^, and 2.5 × 10^−4^ M) in DI water.

Finally, the degree of substitution (DS) of GelMA was calculated using either AM_NMR_ or a combination of the colorimetric results (AM_TNBS_ and AM_Fe(III)_):

$${{\rm{DS}}}_{{\rm{NMR}}}={{\rm{AM}}}_{{\rm{NMR}}}/0.3184\times 100\,( \% )\,{\rm{or}}\,{{\rm{DS}}}_{{\rm{color}}}=({{\rm{AM}}}_{{\rm{TNBS}}}+{{\rm{AM}}}_{{\rm{Fe}}({\rm{III}})})/0.3184\times 100\,( \% ).$$

### Secondary structure of GelMA (DS100_1~5 and DS60_1~5)

To find consistency of secondary structure in GelMA samples, circular dichroism (CD) experiments covering the UV spectral range from 260 to 180 nm, were carried out using Chirascan Plus (Applied Photophysics, Leatherhead, UK). Before every experiment, each sample (0.2 mg mL^−1^ in deionized water) was stored at 4 or 37 °C for 2 h to obtain a stable conformation (triple helix structure or random coil). The acquisitions were performed at 4 or 37 °C after 300 μL of each solution was added in a quartz cell with an optical path length of 1 mm.

### Swelling behavior of bulk GelMA DS100_1~5 and DS60_1~5 hydrogels

GelMA hydrogels (DS100_1~5 and DS60_1~5) for swelling, mechanical and enzymatic degradation testings were conducted as follows. Each GelMA solution (20 (w/v)% in deionized water) containing 0.5 (w/v)% I2959 (prepared at a concentration of 20% as a stock solution in 70% ethanol) was cured under UV light (365 nm with an intensity of 3.5 mW cm^−2^) for 5 min in a polytetrafluoroethylene (PTFE) mold with a diameter of 8 mm and a thickness of 1 mm. Prepared GelMA hydrogels were transferred into a small beaker containing 30 mL of deionized water, incubated for 120 min at an elevated temperature (50 °C) so as to expedite the equilibrium swelling and then weighed at appointed times (5, 10, 15, 20, 30, 40, 50, 60, and 120 min). The swelling degree (*Q*_t_) of GelMA hydrogels at different time points was calculated using the following formula,

$${{\rm{Q}}}_{{\rm{t}}}=\frac{{{\rm{w}}}_{{\rm{t}}}-{{\rm{w}}}_{0}}{{{\rm{w}}}_{0}}\times 100 \% $$

(w_t_: the weight of GelMA hydrogels at t min; w_o_: the dried weight of GelMA hydrogels after freeze-drying at 0 min).

### Mechanical properties of bulk GelMA DS100_1~5 and DS60_1~5 hydrogels

Mechanical properties of GelMA hydrogels with a diameter of 8 mm and a thickness of 1 mm were characterized via sinusoidal shear rheometry. Frequency-sweep measurements were conducted using a rheometer (Discovery Hybrid Rheometer, Thermo, America), equipped with an 8 mm parallel plate (Peltier plate Steel). The storage modulus of each GelMA hydrogel sample was measured at 0.1% strain and 0.1–10 Hz within the viscoelastic range. The running temperature was maintained at 37 °C throughout the measurements.

### Enzymatic degradation of bulk GelMA DS100_1~5 and DS60_1~5 hydrogels

GelMA hydrogels (20 (w/v)%) with a diameter of 8 mm and a thickness of 1 mm were tested for enzymatic degradation. After GelMA hydrogels were placed in deionized water for 3 h at 50 °C to rapidly reach the equilibrium swelling, they were transferred into a new 24-well plate with each well containing 1 mL of 0.05 (v/w)% collagenase (125 CDU mg^−1^, solid) in Hank’s Balanced Salt Solution including 3 mM CaCl_2_. Their enzyme degradation was conducted at 37 °C. Gross images of GelMA hydrogels were taken during the degradation, and mass loss of GelMA hydrogels was also measured.

$${\rm{Mass}}\,{\rm{loss}}=\frac{{{\rm{w}}}_{0}-{{\rm{w}}}_{{\rm{t}}}}{{{\rm{w}}}_{0}}\times 100 \% $$

(w_t_: the weight of each GelMA hydrogel at time t; w_o_: the weight of each GelMA hydrogel at time 0 (the equilibrium swelling).

### Viability of Huh7.5 cells grown on and inside GelMA DS100_1~5 and DS60_1~5 hydrogels

Human hepatocellular carcinoma cells (Huh7.5; ScienCell, Shanghai, China) were cultured in Dulbecco’s Modified Eagle’s Medium with 10% fetal bovine serum and 1% penicillin/streptomycin in a humidified atmosphere at 37 °C with 5% CO_2_. The medium was changed every 3 days. Bulk GelMA (DS100_1~5 and DS60_1~5) hydrogels were prepared in 48-well plates by curing 120 µL of each GelMA solution (20 (w/v)% in PBS) containing 0.5 (w/v)% I2959 in each well. After washing each hydrogel with PBS two times, 500 µL of a medium containing 1 × 10^5^ cells was carefully added to each well coated with each GelMA hydrogel. For cell encapsulation, each GelMA solution (20 (w/v)% in PBS and 0.25 (w/v)% I2959) containing Huh 7.5 cells at a concentration of 3 × 10^6^ cells mL^−1^ was cured in a mold (8 mm in diameter and 1 mm in depth) by exposure to light (365 nm with an intensity of 3.5 mW cm^−2^ for 5 min). Then unencapsulated cells were counted for cell encapsulation efficiency. After 1 day, cell viability on and inside GelMA hydrogels was characterized using Live/Dead® Cell Viability/Cytotoxicity kit. Briefly, 4 µM calcein-acetoxymethyl (calcein-AM) and 8 µM ethidium homodimer-1 (EthD-1) in media were added to each well containing each cell-laden hydrogel, followed by incubation for 1 h at 37 °C. The cytoplasm of live cells and the nuclei of dead cells were stained by calcein-AM (green) and EthD-1 (red), respectively and were observed through a confocal microscope (C2^+^, Nikon, Shanghai, China). Numbers of live and dead cells in three images of each hydrogel were counted using ImageJ. Cell viability was calculated using the following formula,

$${\rm{Cell}}\,{\rm{viability}}\,( \% )=\frac{{\rm{live}}\,{\rm{cells}}}{{\rm{the}}\,{\rm{total}}\,{\rm{number}}\,{\rm{of}}\,{\rm{cells}}}\times 100.$$

### Statistical analysis

Statistical analysis was carried out using the Microsoft Excel^®^ statistical analysis. A one-way ANOVA was used to test for differences among five groups. The standard deviation (s.d.) was calculated and presented for each treatment group. P values below 0.05 were considered statistically significant. The value of n denotes the number of performed samples or the number of independently performed attempts.

## Supplementary information

supporting information
