# Supplementary material for: Gelatin methacryloyl and its hydrogels with an exceptional degree of controllability and batch-to-batch consistency
Source: Sci Rep. 2019 May 3;9:6863. doi: 10.1038/s41598-019-42186-x (PMC6499775; doi:10.1038/s41598-019-42186-x)
Supplement: Supplementary file 1 — supporting information [file 41598_2019_42186_MOESM1_ESM.docx]

**Supporting information**

**Gelatin methacryloyl and its hydrogels with an exceptional degree of controllability and batch-to-batch consistency**

Mengxiang Zhu^1,2^, Yingying Wang ^1^, Gaia Ferracci^3^, Jing Zheng^1,2^, Nam-Joon Cho^3^*, Bae Hoon Lee^1,2^*

1 School of Ophthalmology & Optometry, Eye Hospital, School of Biomedical Engineering, Wenzhou Medical University, Wenzhou, Zhejiang 325027, China

2 Wenzhou Institute of Biomaterials and Engineering, CAS, Wenzhou, Zhejiang 325011, China

3 School of Materials Science and Engineering, Nanyang Technological University, 639798, Singapore

E-mail: [njcho@ntu.edu.sg](mailto:njcho@ntu.edu.sg)

E-mail: bhlee@wibe.ac.cn

Table S1. Summary of some previous GelMA studies showing various reaction parameters, degrees of substitution, and properties.

| **Authors** | **Gelatin**  **type** | **MAA (mL)**  **/**  **gelatin (1 g)** | **Buffer** | *^a^***T**  **(^o^C)** | *^b^***t (h)** | **DS**  **(%)** | *^c^***Mechanical properties**  **(at a concentration)** | **Ref.** |
| --- | --- | --- | --- | --- | --- | --- | --- | --- |
| Nichol  et al. | Type A (300 bloom) | 2 | PBS | 50 | 1 | 81.4 | About 30 kPa (15%)  About 15 kPa (10%)  About 4 kPa (5%)  (compression test) | 1 |
| Pedron et al. | Type A (300 bloom) | 2 | PBS | 60 | 2 | 85 | 55 kPa (10%) (compression test) | 2 |
| Ahadian et al. | Type A (300 bloom) | 0.8 | PBS | 50 | 3 | 80 | 40 kPa (20%) | 3 |
| Schuurman et al. | Type A | 0.58 | PBS | 50 | 1 | 75 | About 180 kPa (20%, crosslinked at 37 ^o^C)  About 250 kPa (20%, crosslinked at 25 ^o^C)  (compression test) | 4 |
| Kaemmerer et al. | Type A | 0.58 | PBS | 50 | 1 | 70-80 | 9 kPa (7%, at 37 ^o^C)  3.7 kPa (5%, at 37 ^o^C)  0.5 kPa (2.5%, at 37 ^o^C)  (compression test) | 5 |
| Kolesky et al. | Type A  (300 bloom) | 0.14 | PBS | 50 | 4 | 50 | 20 kPa  (15%, physical gel at 4 ^o^C) | 6 |
| Sewald et al. | Type A  (233 bloom) | 0.1 | PBS | 37 | 5 | 103 | 6.92 kPa (10%) | 7 |
| Lee et al. | Type A  (175 bloom) | 0.1 | CB/  pH adjustment | 50 | 3 | 97 | 83.2 kPa (30%) | 8 |
| Lee et al. | Type A  (175 bloom) | 0.1 | PBS/  pH adjustment | 50 | 3 | 76.2 | - | 8 |
| Lee et al. | Type A  (175 bloom) | 0.1 | CB | 50 | 1.5 | 94.9 | 67.6 kPa (30%)  34.7 kPa (20%)  9.1 kPa (10%) | 9 |
| Lee et al. | Type B  (225 bloom) | 0.1 | CB | 50 | 1.5 | 94.9 | 147.3 kPa (30%)  53.1 kPa (20%)  10.0 kPa (10%) | 9 |
| Sewald et al. | Type B  (232 bloom) | 0.1 | PBS | 37 | 5 | 103 | 14.40 kPa (10%) | 7 |
| Hoch et al. | Type B  (225 bloom) | 0.08 | PBS | 40-50 | 2 | 67.2 | About 100 kPa  (30%, at 37 ^o^C)  30 kPa (20%, at 37 ^o^C)  7 kPa (10%, at 37 ^o^C) | 10 |
| Billet et al. | Type B  (257 bloom) | 0.0566 | PBS | 40 | 1 | 66 | 107 kPa  (20%, at 37 ^o^C)  14 kPa (10%, at 37 ^o^C)  1.5 kPa (5%, at 37 ^o^C) | 11 |

The symbol of *a* indicates the reaction temperature; *b* indicates the reaction time. *^c^*Mechanical properties of GelMA samples were obtained from rheological measurements unless mentioned. PBS stands for phosphate buffered saline; CB stands for carbonate-bicarbonate (CB) buffer; MAA stands for methacrylic anhydride; DS stands for a degree of substitution.


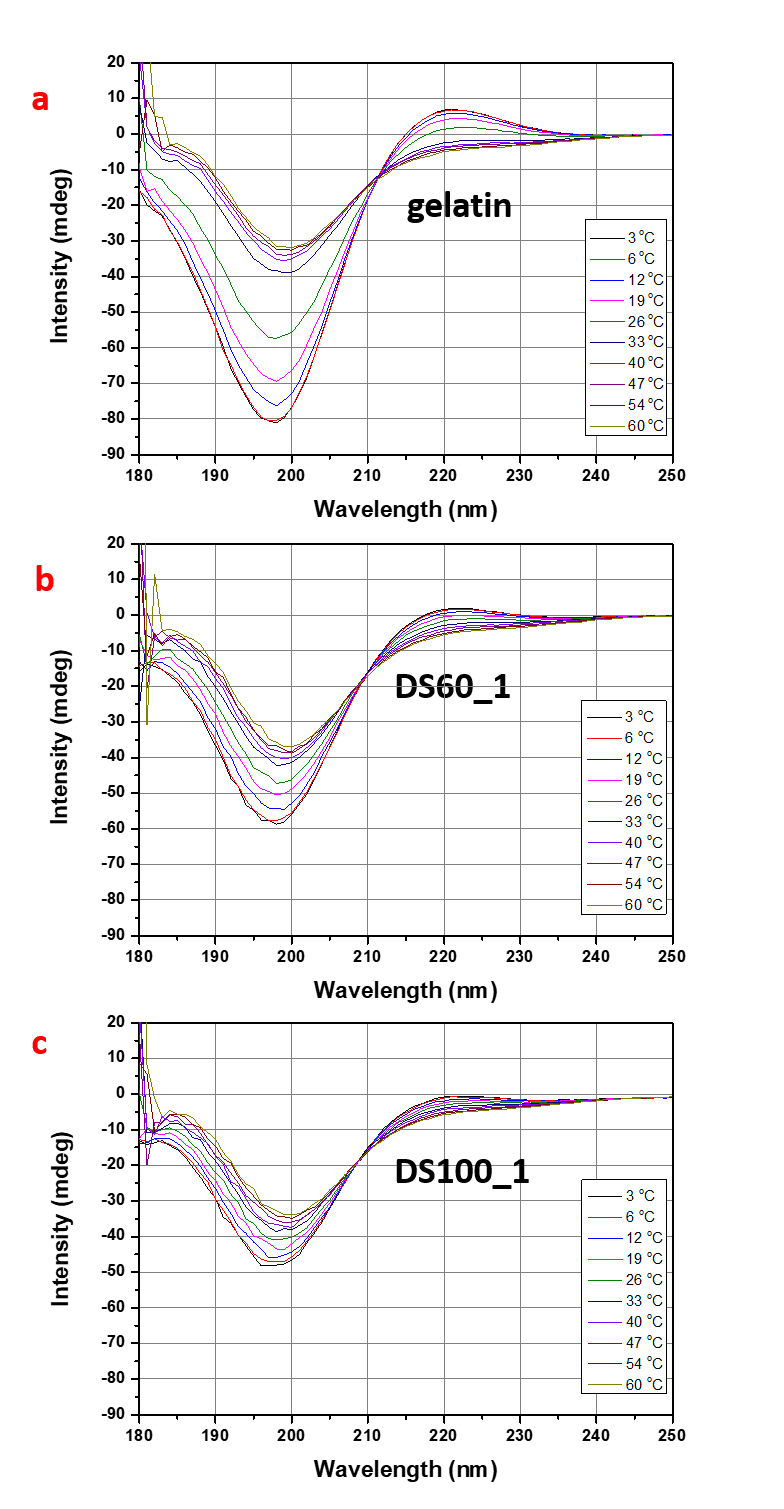


Figure S1. CD spectra of gelatin, DS60_1, and DS100_1 at various temperatures ranging from 3 to 60 ^o^C at 0.25 mg mL^-1^. (a) CD spectra of the gelatin solution. Gelatin exhibited a marked increase in the intensity at 199 nm and a decrease at 222 nm with increasing the temperature, indicating that increasing the temperature could elicit the random coil conformation of gelatin. (b) and (c) CD spectra of DS60_1 and DS100_1. DS60_1 and DS100_1 displayed a higher intensity at 199 nm and a lower intensity at 222 nm, compared with gelatin at low temperature, which means that the methacryloylation of gelatin is the main factor of decreasing the extent of triple-helix formation and increasing the random coil conformation. GelMA with a higher DS (DS100_1) exhibited a less temperature-sensitive phase transition, compare with GelMA with a lower DS (DS60_1) possibly because the high amount of the methacryloylation might impair much of the original gelatin helix structure.

References

1 Nichol, J. W. *et al.* Cell-laden microengineered gelatin methacrylate hydrogels. *Biomaterials* **31**, 5536-5544, doi:10.1016/j.biomaterials.2010.03.064 (2010).

2 Pedron, S., Becka, E. & Harley, B. A. Regulation of glioma cell phenotype in 3D matrices by hyaluronic acid. *Biomaterials* **34**, 7408-7417, doi:10.1016/j.biomaterials.2013.06.024 (2013).

3 Ahadian, S. *et al.* Interdigitated array of Pt electrodes for electrical stimulation and engineering of aligned muscle tissue. *Lab Chip* **12**, 3491-3503, doi:10.1039/c2lc40479f (2012).

4 Schuurman, W. *et al.* Gelatin-methacrylamide hydrogels as potential biomaterials for fabrication of tissue-engineered cartilage constructs. *Macromol Biosci* **13**, 551-561, doi:10.1002/mabi.201200471 (2013).

5 Kaemmerer, E. *et al.* Gelatine methacrylamide-based hydrogels: An alternative three-dimensional cancer cell culture system. *Acta Biomaterialia* **10**, 2551-2562, doi:10.1016/j.actbio.2014.02.035 (2014).

6 Kolesky, D. B. *et al.* 3D Bioprinting of Vascularized, Heterogeneous Cell‐Laden Tissue Constructs. *Advanced Materials* **26**, 3124-3130, doi:10.1002/adma.201305506 (2014).

7 Sewald, L. *et al.* Beyond the Modification Degree: Impact of Raw Material on Physicochemical Properties of Gelatin Type A and Type B Methacryloyls. *Macromol Biosci* **18**, e1800168, doi:10.1002/mabi.201800168 (2018).

8 Lee, B. H., Shirahama, H., Cho, N.-J. & Tan, L. P. Efficient and controllable synthesis of highly substituted gelatin methacrylamide for mechanically stiff hydrogels. *RSC Advances* **5**, 106094-106097, doi:10.1039/c5ra22028a (2015).

9 Lee, B. H., Lum, N., Seow, L. Y., Lim, P. Q. & Tan, L. P. Synthesis and Characterization of Types A and B Gelatin Methacryloyl for Bioink Applications. *Materials* **9**, doi:10.3390/ma9100797 (2016).

10 Hoch, E., Schuh, C., Hirth, T., Tovar, G. E. & Borchers, K. Stiff gelatin hydrogels can be photo-chemically synthesized from low viscous gelatin solutions using molecularly functionalized gelatin with a high degree of methacrylation. *J Mater Sci Mater Med* **23**, 2607-2617, doi:10.1007/s10856-012-4731-2 (2012).

11 Billiet, T. *et al.* Quantitative contrasts in the photopolymerization of acrylamide and methacrylamide-functionalized gelatin hydrogel building blocks. *Macromol Biosci* **13**, 1531-1545, doi:10.1002/mabi.201300143 (2013).
